# 4-(5-{2-[5-(4-Cyano­phen­yl)-3-methyl­thio­phen-2-yl]-3,3,4,4,5,5-hexa­fluoro­cyclo­pent-1-en-1-yl}-4-methyl­thio­phen-2-yl)benzo­nitrile chloro­form hemisolvate

**DOI:** 10.1107/S1600536813014852

**Published:** 2013-06-08

**Authors:** Gamal A. El-Hiti, Keith Smith, Ali Masmali, Asim A. Balakit, Benson M. Kariuki

**Affiliations:** a Department of Optometry, College of Applied Medical Sciences, King Saud University, PO Box 10219, Riyadh 11433, Saudi Arabia; b School of Chemistry, Cardiff University, Main Building, Park Place, Cardiff CF10 3AT, Wales

## Abstract

The crystal structure of the title compound, C_29_H_16_F_6_N_2_S_2_·0.5CHCl_3_, consists of mol­ecules with disordered perfluoro­cyclo­pentene rings [occupancy ratio 0.685 (3):0.315 (3)] and close F⋯F contacts (in the range 2.45–2.73 Å) between mol­ecules. The short contacts are associated with the disorder. The dihedral angle between thiophene rings is 57.44 (8)°. The 5-(4-cyano­phen­yl)-3-methyl-2-thienyl groups of adjacent mol­ecules are parallel, leading to zigzag chains of mol­ecules along [101]. The dihedral angles between each thiophene ring and its adjacent cyanobenzene ring are 8.9 (2) and 7.15 (10)°.

## Related literature

For applications of substituted thienylperfluoro­cyclo­pentenes as switches, see: Waldeck (1991 ▶); Pu *et al.* (2006 ▶); Dulic *et al.* (2007 ▶). For related structures, see: Irie *et al.* (1995 ▶, 2000 ▶); Morimitsu *et al.* (2002 ▶); Mori *et al.* (2011 ▶). 
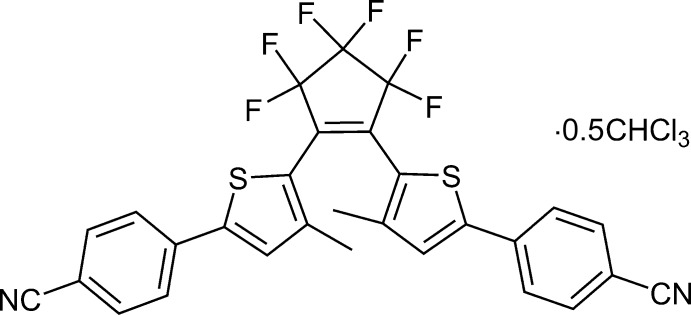


## Experimental

### 

#### Crystal data

C_29_H_16_F_6_N_2_S_2_·0.5CHCl_3_
*M* *_r_* = 630.24
Monoclinic, 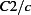

*a* = 18.4237 (4) Å
*b* = 15.7594 (6) Å
*c* = 20.9299 (7) Å
β = 113.280 (2)°
*V* = 5582.2 (3) Å^3^
*Z* = 8
Mo *K*α radiation
μ = 0.40 mm^−1^
*T* = 150 K
0.40 × 0.30 × 0.30 mm

#### Data collection

Nonius KappaCCD diffractometer
Absorption correction: multi-scan (*DENZO*/*SCALEPACK*; Otwinowski & Minor, 1997 ▶) *T*_min_ = 0.857, *T*_max_ = 0.890
10636 measured reflections
6329 independent reflections
4168 reflections with *I* > 2σ(*I*)
*R*_int_ = 0.042

#### Refinement

*R*[*F* ^2^ > 2σ(*F* ^2^)] = 0.069
*wR*(*F* ^2^) = 0.174
*S* = 1.04
6329 reflections
455 parameters
92 restraints
H-atom parameters constrained
Δρ_max_ = 0.32 e Å^−3^
Δρ_min_ = −0.42 e Å^−3^

### 

Data collection: *COLLECT* (Nonius, 2000 ▶); cell refinement: *DENZO*/*SCALEPACK* (Otwinowski & Minor, 1997 ▶); data reduction: *DENZO*/*SCALEPACK*; program(s) used to solve structure: *SHELXS97* (Sheldrick, 2008 ▶); program(s) used to refine structure: *SHELXL97* (Sheldrick, 2008 ▶); molecular graphics: *ORTEP99* for Windows (Farrugia, 2012 ▶); software used to prepare material for publication: *WinGX* (Farrugia, 2012 ▶) and *CHEMDRAW Ultra* (Cambridge Soft, 2001 ▶).

## Supplementary Material

Crystal structure: contains datablock(s) I, global. DOI: 10.1107/S1600536813014852/mw2111sup1.cif

Structure factors: contains datablock(s) I. DOI: 10.1107/S1600536813014852/mw2111Isup2.hkl

Supplementary material file. DOI: 10.1107/S1600536813014852/mw2111Isup3.cml

Additional supplementary materials:  crystallographic information; 3D view; checkCIF report

## Figures and Tables

**Fig. 1.**
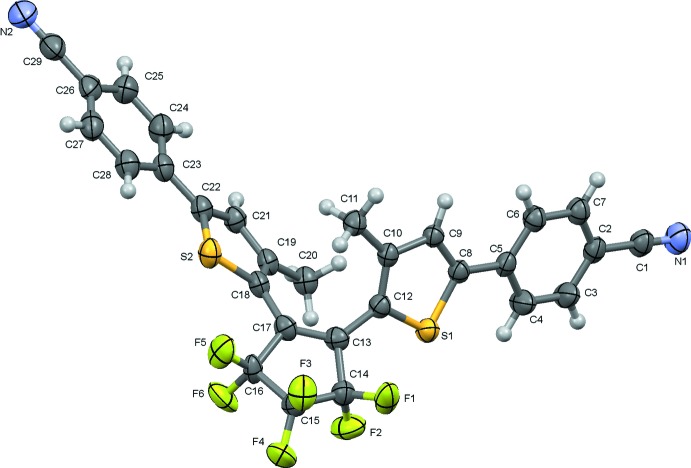
A molecule of I showing the major component of the disorder with atom labels and 50% probability displacement ellipsoids for non-H atoms.

**Fig. 2.**
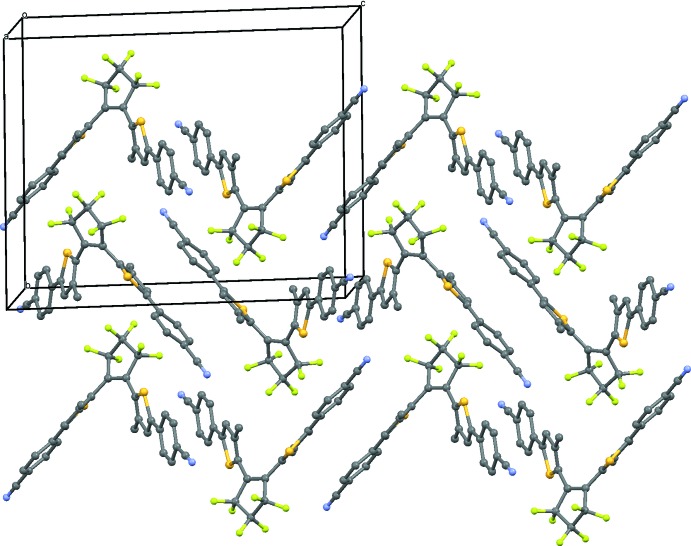
A segment of the crystal structure of I showing the parallel arrangement of 5-(4-cyanophenyl)-3-methyl-2-thienyl groups with the minor component of the disorder and hydrogen atoms omitted.

## supplementary crystallographic information

### Comment

During research focused on new synthetic routes towards novel substituted thienylperfluorocyclopentene, we have synthesized and purified the title compound (I). The crystal structure of I contains disordered chloroform solvent located close to a two-fold axis. The perfluorocyclopentene ring is disordered with two components having 68.5 (3)% and 31.5 (3)% occupancy related by a flip between alternative envelope configurations (Figure 2). Unfavourably close F···F contacts with distances in the range 2.45–2.73 Å between pairs of molecules may partly account for the disorder in the ring. The planes of both 5-(4-cyanophenyl)-3-methyl-2-thienyl groups of the molecule are parallel with similar groups of neighbouring molecules, with interplanar distances of 3.39 Å and 3.50 Å, forming zigzag chains parallel to the [101] direction (Figure 2). Stacking of the chains forms planes parallel to the (101) plane.

### Refinement

H atoms were positioned geometrically and refined using a riding model with *U*_iso_(H) = 1.2 times *U*_eq_ for the associated atom (1.5 times for methyl groups with free rotation about the C—C bond). The perfluorocyclopentene ring is disordered with two components. Refinement of the disorder was performed using PART 1, PART 2 and FVAR in *SHELX*. The minor component was restrained using the SAME instruction in *SHELX* to give similar bond distances and angles as the major component. Related atoms and atoms in close proximity were refined with identical or similar displacement parameters using either EADP or SIMU instructions in *SHELX*. The chloroform site is also disordered and contains half of the molecules with two unique components close to a symmetry element. The geometry of the major component of disordered chloform was restrained and the second component was constrained to the same geometry.

### Crystal data

**Table:**

C_29_H_16_F_6_N_2_S_2_·0.5CHCl_3_	*F*(000) = 2552
*M**_r_* = 630.24	*D*_x_ = 1.500 Mg m^−^^3^
Monoclinic, *C*2/*c*	Mo *K*α radiation, λ = 0.71073 Å
Hall symbol: -C 2yc	Cell parameters from 4168 reflections
*a* = 18.4237 (4) Å	θ = 2.8–27.4°
*b* = 15.7594 (6) Å	µ = 0.40 mm^−^^1^
*c* = 20.9299 (7) Å	*T* = 150 K
β = 113.280 (2)°	Block, yellow
*V* = 5582.2 (3) Å^3^	0.40 × 0.30 × 0.30 mm
*Z* = 8	

### Data collection

**Table:**

Nonius KappaCCD diffractometer	6329 independent reflections
Radiation source: fine-focus sealed tube	4168 reflections with *I* > 2σ(*I*)
Graphite monochromator	*R*_int_ = 0.042
CCD slices scans	θ_max_ = 27.4°, θ_min_ = 2.8°
Absorption correction: multi-scan (*DENZO*/*SCALEPACK*; Otwinowski & Minor, 1997)	*h* = −23→23
*T*_min_ = 0.857, *T*_max_ = 0.890	*k* = −18→20
10636 measured reflections	*l* = −27→27

### Refinement

**Table:**

Refinement on *F*^2^	Primary atom site location: structure-invariant direct methods
Least-squares matrix: full	Secondary atom site location: difference Fourier map
*R*[*F*^2^ > 2σ(*F*^2^)] = 0.069	Hydrogen site location: inferred from neighbouring sites
*wR*(*F*^2^) = 0.174	H-atom parameters constrained
*S* = 1.04	*w* = 1/[σ^2^(*F*_o_^2^) + (0.0749*P*)^2^ + 7.3013*P*] where *P* = (*F*_o_^2^ + 2*F*_c_^2^)/3
6329 reflections	(Δ/σ)_max_ = 0.001
455 parameters	Δρ_max_ = 0.32 e Å^−^^3^
92 restraints	Δρ_min_ = −0.42 e Å^−^^3^

### Special details

**Table:**

Geometry. All s.u.'s (except the s.u. in the dihedral angle between two l.s. planes) are estimated using the full covariance matrix. The cell s.u.'s are taken into account individually in the estimation of s.u.'s in distances, angles and torsion angles; correlations between s.u.'s in cell parameters are only used when they are defined by crystal symmetry. An approximate (isotropic) treatment of cell s.u.'s is used for estimating s.u.'s involving l.s. planes.
Refinement. Refinement of *F*^2^ against ALL reflections. The weighted *R*-factor *wR* and goodness of fit *S* are based on *F*^2^, conventional *R*-factors *R* are based on *F*, with *F* set to zero for negative *F*^2^. The threshold expression of *F*^2^ > 2σ(*F*^2^) is used only for calculating *R*-factors(gt) *etc*. and is not relevant to the choice of reflections for refinement. *R*-factors based on *F*^2^ are statistically about twice as large as those based on *F*, and *R*- factors based on ALL data will be even larger.

### Fractional atomic coordinates and isotropic or equivalent isotropic displacement parameters (Å^2^)

**Table:**

	*x*	*y*	*z*	*U*_iso_*/*U*_eq_	Occ. (<1)
C1	0.33279 (18)	1.2412 (2)	−0.04232 (18)	0.0504 (8)	
C2	0.39449 (16)	1.18251 (19)	−0.00118 (17)	0.0445 (7)	
C3	0.38411 (18)	1.1334 (2)	0.04903 (19)	0.0572 (9)	
H3	0.3372	1.1388	0.0573	0.069*	
C4	0.44183 (18)	1.0763 (2)	0.0874 (2)	0.0555 (9)	
H4	0.4340	1.0422	0.1215	0.067*	
C5	0.51131 (16)	1.06837 (18)	0.07655 (15)	0.0395 (7)	
C6	0.52104 (17)	1.11841 (19)	0.02572 (16)	0.0440 (7)	
H6	0.5679	1.1131	0.0174	0.053*	
C7	0.46373 (17)	1.1756 (2)	−0.01281 (17)	0.0461 (7)	
H7	0.4714	1.2099	−0.0470	0.055*	
C8	0.57310 (16)	1.00838 (18)	0.11772 (15)	0.0375 (6)	
C9	0.64811 (15)	1.00024 (18)	0.12016 (14)	0.0356 (6)	
H9	0.6676	1.0340	0.0928	0.043*	
C10	0.69445 (15)	0.93707 (17)	0.16712 (15)	0.0357 (6)	
C11	0.77772 (17)	0.9166 (2)	0.17704 (17)	0.0457 (7)	
H11A	0.8145	0.9497	0.2161	0.069*	
H11B	0.7855	0.9309	0.1346	0.069*	
H11C	0.7876	0.8560	0.1869	0.069*	
C12	0.65256 (16)	0.89691 (18)	0.19993 (15)	0.0379 (6)	
C13	0.68037 (16)	0.82944 (18)	0.25213 (16)	0.0405 (7)	0.685 (3)
C14	0.6335 (5)	0.7514 (7)	0.2517 (3)	0.0471 (18)	0.685 (3)
C15	0.6940 (3)	0.6865 (4)	0.2969 (3)	0.0438 (13)	0.685 (3)
C16	0.7615 (4)	0.7421 (5)	0.3474 (3)	0.0413 (15)	0.685 (3)
C17	0.74912 (17)	0.82530 (18)	0.30905 (16)	0.0418 (7)	0.685 (3)
F1	0.5924 (3)	0.7238 (2)	0.1859 (2)	0.0739 (14)	0.685 (3)
F2	0.5788 (2)	0.7682 (2)	0.2782 (3)	0.0744 (11)	0.685 (3)
F3	0.71811 (19)	0.6400 (2)	0.25617 (17)	0.0661 (10)	0.685 (3)
F4	0.6623 (3)	0.6336 (3)	0.3290 (2)	0.0727 (13)	0.685 (3)
F5	0.83090 (19)	0.7038 (2)	0.3585 (2)	0.0710 (11)	0.685 (3)
F6	0.7555 (4)	0.7454 (4)	0.4099 (3)	0.0715 (15)	0.685 (3)
C13A	0.68037 (16)	0.82944 (18)	0.25213 (16)	0.0405 (7)	0.315 (3)
C14A	0.6303 (12)	0.7494 (16)	0.2320 (11)	0.0471 (18)	0.315 (3)
C15A	0.6662 (8)	0.6950 (10)	0.2968 (9)	0.0438 (13)	0.315 (3)
C16A	0.7502 (10)	0.7307 (12)	0.3286 (10)	0.0413 (15)	0.315 (3)
C17A	0.74912 (17)	0.82530 (18)	0.30905 (16)	0.0418 (7)	0.315 (3)
F1A	0.6337 (5)	0.7071 (6)	0.1786 (5)	0.0739 (14)	0.315 (3)
F2A	0.5523 (4)	0.7582 (5)	0.2185 (7)	0.079 (3)	0.315 (3)
F3A	0.6725 (7)	0.6131 (6)	0.2876 (7)	0.107 (4)	0.315 (3)
F4A	0.6312 (5)	0.7076 (7)	0.3410 (4)	0.099 (3)	0.315 (3)
F5A	0.8056 (5)	0.6908 (5)	0.3168 (5)	0.0710 (11)	0.315 (3)
F6A	0.7809 (10)	0.7365 (11)	0.4001 (8)	0.0715 (15)	0.315 (3)
C18	0.80784 (17)	0.89096 (19)	0.33911 (15)	0.0399 (7)	
C19	0.79551 (17)	0.97566 (19)	0.34710 (14)	0.0398 (7)	
C20	0.71654 (17)	1.0172 (2)	0.33051 (16)	0.0456 (7)	
H20A	0.6964	1.0395	0.2829	0.068*	
H20B	0.7225	1.0639	0.3632	0.068*	
H20C	0.6793	0.9754	0.3346	0.068*	
C21	0.86700 (17)	1.0205 (2)	0.37701 (15)	0.0427 (7)	
H21	0.8692	1.0796	0.3864	0.051*	
C22	0.93311 (18)	0.9721 (2)	0.39144 (15)	0.0444 (7)	
C23	1.01629 (18)	0.9987 (2)	0.42181 (15)	0.0448 (7)	
C24	1.0359 (2)	1.0850 (2)	0.43260 (18)	0.0566 (9)	
H24	0.9945	1.1257	0.4198	0.068*	
C25	1.11288 (19)	1.1127 (2)	0.46109 (18)	0.0548 (9)	
H25	1.1244	1.1715	0.4679	0.066*	
C26	1.17385 (19)	1.0532 (2)	0.47980 (16)	0.0506 (8)	
C27	1.15609 (19)	0.9674 (3)	0.46981 (17)	0.0564 (9)	
H27	1.1977	0.9270	0.4828	0.068*	
C28	1.07840 (19)	0.9403 (2)	0.44115 (17)	0.0529 (8)	
H28	1.0671	0.8813	0.4346	0.063*	
C29	1.2554 (2)	1.0801 (2)	0.50949 (19)	0.0578 (9)	
N1	0.28399 (17)	1.28672 (19)	−0.07370 (17)	0.0657 (9)	
N2	1.32050 (18)	1.1008 (2)	0.53249 (18)	0.0706 (9)	
S1	0.55727 (4)	0.93605 (5)	0.17304 (4)	0.0422 (2)	
S2	0.90768 (5)	0.86753 (5)	0.36945 (4)	0.0485 (2)	
C30	0.4976 (5)	1.2497 (5)	0.2286 (6)	0.057 (3)	0.334 (3)
H30	0.4817	1.2508	0.1771	0.068*	0.334 (3)
Cl1	0.4299 (2)	1.1883 (3)	0.2487 (3)	0.0696 (8)	0.334 (3)
Cl2	0.5063 (6)	1.3539 (2)	0.2602 (6)	0.075 (3)	0.334 (3)
Cl3	0.5920 (2)	1.2059 (3)	0.2688 (4)	0.0696 (8)	0.334 (3)
C30A	0.5104 (14)	1.2114 (16)	0.2263 (15)	0.057 (3)	0.166 (3)
H30A	0.5259	1.1925	0.1879	0.068*	0.166 (3)
Cl1A	0.4634 (6)	1.1251 (7)	0.2494 (4)	0.116 (4)	0.166 (3)
Cl2A	0.4406 (6)	1.2868 (9)	0.1950 (5)	0.121 (4)	0.166 (3)
Cl3A	0.5987 (5)	1.2325 (6)	0.3015 (6)	0.0696 (8)	0.166 (3)

### Atomic displacement parameters (Å^2^)

**Table:**

	*U*^11^	*U*^22^	*U*^33^	*U*^12^	*U*^13^	*U*^23^
C1	0.0403 (17)	0.0414 (17)	0.059 (2)	0.0019 (13)	0.0083 (15)	0.0036 (16)
C2	0.0359 (15)	0.0356 (15)	0.0499 (18)	0.0013 (12)	0.0039 (13)	−0.0001 (14)
C3	0.0355 (16)	0.057 (2)	0.075 (2)	0.0085 (14)	0.0182 (16)	0.0234 (19)
C4	0.0401 (17)	0.058 (2)	0.069 (2)	0.0099 (14)	0.0219 (16)	0.0233 (18)
C5	0.0324 (14)	0.0359 (15)	0.0403 (16)	−0.0001 (11)	0.0038 (12)	−0.0028 (13)
C6	0.0415 (16)	0.0406 (16)	0.0455 (18)	0.0043 (12)	0.0125 (13)	0.0021 (14)
C7	0.0441 (16)	0.0406 (16)	0.0467 (18)	0.0046 (13)	0.0106 (14)	0.0027 (14)
C8	0.0367 (14)	0.0335 (14)	0.0364 (15)	−0.0012 (11)	0.0082 (12)	0.0001 (12)
C9	0.0353 (14)	0.0338 (14)	0.0334 (15)	−0.0013 (11)	0.0090 (11)	0.0006 (12)
C10	0.0341 (14)	0.0334 (14)	0.0345 (15)	0.0017 (11)	0.0081 (11)	−0.0023 (12)
C11	0.0399 (16)	0.0509 (18)	0.0444 (18)	0.0042 (13)	0.0146 (13)	0.0060 (15)
C12	0.0362 (14)	0.0330 (14)	0.0413 (16)	0.0020 (11)	0.0119 (12)	0.0004 (13)
C13	0.0418 (15)	0.0337 (15)	0.0469 (17)	0.0043 (12)	0.0185 (13)	0.0029 (13)
C14	0.0419 (19)	0.0447 (19)	0.048 (5)	−0.0011 (15)	0.011 (3)	0.005 (4)
C15	0.039 (4)	0.037 (2)	0.056 (2)	0.006 (3)	0.019 (3)	0.0123 (19)
C16	0.050 (3)	0.033 (3)	0.027 (5)	0.003 (2)	0.000 (3)	−0.004 (3)
C17	0.0467 (16)	0.0349 (15)	0.0424 (17)	0.0081 (12)	0.0160 (13)	0.0056 (13)
F1	0.067 (3)	0.0438 (19)	0.0713 (19)	−0.016 (2)	−0.014 (3)	0.0090 (16)
F2	0.0487 (19)	0.067 (2)	0.115 (3)	0.0030 (15)	0.041 (2)	0.021 (2)
F3	0.072 (2)	0.0518 (19)	0.062 (2)	0.0090 (16)	0.0134 (16)	−0.0098 (16)
F4	0.075 (2)	0.061 (3)	0.073 (3)	−0.0102 (19)	0.021 (2)	0.029 (2)
F5	0.050 (2)	0.0415 (15)	0.096 (3)	0.0148 (14)	0.0020 (18)	0.016 (2)
F6	0.113 (5)	0.063 (2)	0.038 (2)	−0.009 (3)	0.0298 (19)	0.0121 (17)
C13A	0.0418 (15)	0.0337 (15)	0.0469 (17)	0.0043 (12)	0.0185 (13)	0.0029 (13)
C14A	0.0419 (19)	0.0447 (19)	0.048 (5)	−0.0011 (15)	0.011 (3)	0.005 (4)
C15A	0.039 (4)	0.037 (2)	0.056 (2)	0.006 (3)	0.019 (3)	0.0123 (19)
C16A	0.050 (3)	0.033 (3)	0.027 (5)	0.003 (2)	0.000 (3)	−0.004 (3)
C17A	0.0467 (16)	0.0349 (15)	0.0424 (17)	0.0081 (12)	0.0160 (13)	0.0056 (13)
F1A	0.067 (3)	0.0438 (19)	0.0713 (19)	−0.016 (2)	−0.014 (3)	0.0090 (16)
F2A	0.054 (4)	0.060 (5)	0.122 (8)	−0.001 (4)	0.033 (5)	0.030 (5)
F3A	0.111 (8)	0.038 (5)	0.130 (10)	−0.001 (4)	0.003 (8)	0.027 (6)
F4A	0.102 (6)	0.114 (8)	0.089 (6)	−0.007 (5)	0.044 (5)	0.047 (6)
F5A	0.050 (2)	0.0415 (15)	0.096 (3)	0.0148 (14)	0.0020 (18)	0.016 (2)
F6A	0.113 (5)	0.063 (2)	0.038 (2)	−0.009 (3)	0.0298 (19)	0.0121 (17)
C18	0.0443 (15)	0.0399 (15)	0.0312 (15)	0.0080 (12)	0.0103 (12)	0.0057 (13)
C19	0.0456 (16)	0.0416 (16)	0.0285 (15)	0.0059 (12)	0.0107 (12)	0.0039 (13)
C20	0.0492 (17)	0.0432 (17)	0.0415 (17)	0.0076 (13)	0.0149 (14)	−0.0022 (14)
C21	0.0492 (17)	0.0408 (16)	0.0345 (16)	0.0019 (13)	0.0127 (13)	−0.0022 (13)
C22	0.0482 (17)	0.0474 (18)	0.0333 (16)	0.0030 (14)	0.0115 (13)	0.0001 (14)
C23	0.0472 (17)	0.0513 (18)	0.0320 (15)	0.0049 (14)	0.0115 (13)	0.0020 (14)
C24	0.0515 (19)	0.060 (2)	0.050 (2)	0.0063 (16)	0.0107 (15)	0.0051 (17)
C25	0.0503 (19)	0.059 (2)	0.049 (2)	−0.0030 (15)	0.0135 (15)	0.0018 (17)
C26	0.0487 (18)	0.072 (2)	0.0309 (16)	−0.0037 (16)	0.0156 (13)	0.0032 (16)
C27	0.0465 (18)	0.078 (3)	0.0416 (19)	0.0094 (17)	0.0141 (15)	0.0068 (18)
C28	0.0550 (19)	0.058 (2)	0.0430 (19)	0.0073 (15)	0.0168 (15)	−0.0004 (16)
C29	0.054 (2)	0.072 (2)	0.050 (2)	0.0061 (17)	0.0234 (17)	0.0149 (18)
N1	0.0491 (16)	0.0540 (18)	0.079 (2)	0.0116 (14)	0.0094 (15)	0.0143 (17)
N2	0.0505 (18)	0.092 (3)	0.069 (2)	−0.0019 (17)	0.0233 (15)	0.0148 (19)
S1	0.0354 (4)	0.0395 (4)	0.0490 (5)	0.0026 (3)	0.0139 (3)	0.0064 (3)
S2	0.0460 (4)	0.0449 (4)	0.0457 (5)	0.0101 (3)	0.0088 (3)	0.0021 (4)
C30	0.036 (5)	0.083 (10)	0.055 (6)	0.003 (7)	0.020 (5)	−0.004 (7)
Cl1	0.0468 (17)	0.075 (2)	0.079 (3)	−0.0058 (11)	0.016 (2)	0.0163 (16)
Cl2	0.100 (4)	0.0733 (19)	0.081 (7)	−0.038 (4)	0.067 (5)	−0.031 (4)
Cl3	0.0468 (17)	0.075 (2)	0.079 (3)	−0.0058 (11)	0.016 (2)	0.0163 (16)
C30A	0.036 (5)	0.083 (10)	0.055 (6)	0.003 (7)	0.020 (5)	−0.004 (7)
Cl1A	0.115 (6)	0.125 (8)	0.080 (5)	−0.071 (6)	0.009 (5)	0.016 (6)
Cl2A	0.086 (6)	0.175 (10)	0.110 (6)	0.069 (7)	0.048 (5)	0.042 (7)
Cl3A	0.0468 (17)	0.075 (2)	0.079 (3)	−0.0058 (11)	0.016 (2)	0.0163 (16)

### Geometric parameters (Å, º)

**Table:**

C1—N1	1.135 (4)	C14A—C15A	1.52 (2)
C1—C2	1.454 (4)	C15A—F3A	1.316 (18)
C2—C3	1.378 (5)	C15A—F4A	1.335 (17)
C2—C7	1.394 (5)	C15A—C16A	1.53 (2)
C3—C4	1.382 (4)	C16A—F5A	1.303 (19)
C3—H3	0.9500	C16A—F6A	1.378 (18)
C4—C5	1.391 (4)	C18—C19	1.375 (4)
C4—H4	0.9500	C18—S2	1.732 (3)
C5—C6	1.392 (4)	C19—C21	1.405 (4)
C5—C8	1.469 (4)	C19—C20	1.506 (4)
C6—C7	1.380 (4)	C20—H20A	0.9800
C6—H6	0.9500	C20—H20B	0.9800
C7—H7	0.9500	C20—H20C	0.9800
C8—C9	1.369 (4)	C21—C22	1.365 (4)
C8—S1	1.730 (3)	C21—H21	0.9500
C9—C10	1.422 (4)	C22—C23	1.469 (4)
C9—H9	0.9500	C22—S2	1.726 (3)
C10—C12	1.374 (4)	C23—C28	1.398 (4)
C10—C11	1.500 (4)	C23—C24	1.402 (5)
C11—H11A	0.9800	C24—C25	1.375 (5)
C11—H11B	0.9800	C24—H24	0.9500
C11—H11C	0.9800	C25—C26	1.395 (5)
C12—C13	1.464 (4)	C25—H25	0.9500
C12—S1	1.732 (3)	C26—C27	1.387 (5)
C13—C17	1.355 (4)	C26—C29	1.444 (5)
C13—C14	1.500 (10)	C27—C28	1.383 (5)
C14—F2	1.355 (7)	C27—H27	0.9500
C14—F1	1.356 (7)	C28—H28	0.9500
C14—C15	1.534 (10)	C29—N2	1.149 (4)
C15—F3	1.327 (6)	C30—Cl3	1.748 (8)
C15—F4	1.341 (6)	C30—Cl2	1.754 (8)
C15—C16	1.547 (8)	C30—Cl1	1.755 (8)
C16—F5	1.349 (7)	C30—H30	1.0000
C16—F6	1.355 (6)	C30A—Cl2A	1.68 (2)
C16—C17	1.507 (8)	C30A—Cl1A	1.78 (3)
C17—C18	1.449 (4)	C30A—Cl3A	1.79 (3)
C14A—F1A	1.32 (2)	C30A—H30A	1.0000
C14A—F2A	1.36 (2)		
			
N1—C1—C2	178.8 (4)	F1A—C14A—C15A	108.6 (18)
C3—C2—C7	120.1 (3)	F2A—C14A—C15A	107.2 (16)
C3—C2—C1	119.8 (3)	F3A—C15A—F4A	110.0 (14)
C7—C2—C1	120.1 (3)	F3A—C15A—C14A	117.1 (17)
C2—C3—C4	120.1 (3)	F4A—C15A—C14A	112.0 (13)
C2—C3—H3	119.9	F3A—C15A—C16A	106.9 (12)
C4—C3—H3	119.9	F4A—C15A—C16A	109.1 (14)
C3—C4—C5	120.7 (3)	C14A—C15A—C16A	100.9 (14)
C3—C4—H4	119.7	F5A—C16A—F6A	102.8 (14)
C5—C4—H4	119.7	F5A—C16A—C15A	118.3 (13)
C4—C5—C6	118.6 (3)	F6A—C16A—C15A	114.0 (18)
C4—C5—C8	120.8 (3)	C19—C18—C17	128.0 (3)
C6—C5—C8	120.6 (3)	C19—C18—S2	111.4 (2)
C7—C6—C5	121.1 (3)	C17—C18—S2	120.6 (2)
C7—C6—H6	119.4	C18—C19—C21	111.8 (3)
C5—C6—H6	119.4	C18—C19—C20	125.8 (3)
C6—C7—C2	119.4 (3)	C21—C19—C20	122.3 (3)
C6—C7—H7	120.3	C19—C20—H20A	109.5
C2—C7—H7	120.3	C19—C20—H20B	109.5
C9—C8—C5	128.3 (3)	H20A—C20—H20B	109.5
C9—C8—S1	110.4 (2)	C19—C20—H20C	109.5
C5—C8—S1	121.4 (2)	H20A—C20—H20C	109.5
C8—C9—C10	114.4 (3)	H20B—C20—H20C	109.5
C8—C9—H9	122.8	C22—C21—C19	114.6 (3)
C10—C9—H9	122.8	C22—C21—H21	122.7
C12—C10—C9	111.4 (2)	C19—C21—H21	122.7
C12—C10—C11	125.3 (3)	C21—C22—C23	128.6 (3)
C9—C10—C11	123.2 (3)	C21—C22—S2	110.4 (2)
C10—C11—H11A	109.5	C23—C22—S2	121.0 (2)
C10—C11—H11B	109.5	C28—C23—C24	117.6 (3)
H11A—C11—H11B	109.5	C28—C23—C22	122.2 (3)
C10—C11—H11C	109.5	C24—C23—C22	120.3 (3)
H11A—C11—H11C	109.5	C25—C24—C23	122.2 (3)
H11B—C11—H11C	109.5	C25—C24—H24	118.9
C10—C12—C13	127.4 (3)	C23—C24—H24	118.9
C10—C12—S1	111.8 (2)	C24—C25—C26	119.1 (3)
C13—C12—S1	120.8 (2)	C24—C25—H25	120.4
C17—C13—C12	128.4 (3)	C26—C25—H25	120.4
C17—C13—C14	107.5 (4)	C27—C26—C25	119.8 (3)
C12—C13—C14	124.0 (4)	C27—C26—C29	119.7 (3)
F2—C14—F1	105.7 (6)	C25—C26—C29	120.5 (3)
F2—C14—C13	111.0 (7)	C28—C27—C26	120.6 (3)
F1—C14—C13	111.0 (6)	C28—C27—H27	119.7
F2—C14—C15	110.7 (6)	C26—C27—H27	119.7
F1—C14—C15	113.0 (7)	C27—C28—C23	120.7 (3)
C13—C14—C15	105.5 (5)	C27—C28—H28	119.7
F3—C15—F4	107.5 (5)	C23—C28—H28	119.7
F3—C15—C14	108.5 (5)	N2—C29—C26	179.0 (5)
F4—C15—C14	111.7 (5)	C8—S1—C12	92.00 (14)
F3—C15—C16	111.7 (4)	C22—S2—C18	91.79 (15)
F4—C15—C16	113.7 (5)	Cl3—C30—Cl2	105.3 (6)
C14—C15—C16	103.6 (5)	Cl3—C30—Cl1	109.4 (5)
F5—C16—F6	106.5 (5)	Cl2—C30—Cl1	113.2 (6)
F5—C16—C17	114.5 (6)	Cl3—C30—H30	109.6
F6—C16—C17	115.4 (5)	Cl2—C30—H30	109.6
F5—C16—C15	108.3 (5)	Cl1—C30—H30	109.6
F6—C16—C15	109.3 (6)	Cl2A—C30A—Cl1A	105.5 (13)
C17—C16—C15	102.6 (4)	Cl2A—C30A—Cl3A	120.0 (16)
C13—C17—C18	128.7 (3)	Cl1A—C30A—Cl3A	105.9 (15)
C13—C17—C16	113.7 (3)	Cl2A—C30A—H30A	108.3
C18—C17—C16	117.4 (3)	Cl1A—C30A—H30A	108.3
F1A—C14A—F2A	106.0 (15)	Cl3A—C30A—H30A	108.3
